# “Consent Does Not Scale”: Laying Out the Tensions in Balancing Patient Autonomy with Public Benefit in Commercializing Biospecimens

**DOI:** 10.1017/jme.2023.74

**Published:** 2023

**Authors:** Kayte Spector-Bagdady

**Affiliations:** 1. UNIVERSITY OF MICHIGAN, ANN ARBOR, MI, USA

**Keywords:** Biospecimen, Biobank, Commercialization, Research

Selling Clinical Biospecimens: Guidance for Researchers and Private Industry” by Peter Schwartz and Jane Hartsock explores the important and complex world of academic medical centers (AMCs) commercializing patient biospecimens.^1^ It is important because specimen commercialization can enable critical research. It is complex because it makes most patients uncomfortable. As the authors point out, there are few legal requirements to structure options. Striking the right balance is left up to individual policies and procedures on a hospital by hospital (or even doctor by doctor) basis.

Collaborations between AMCs and commercial industry can fund and accelerate important health research with biospecimens and related phenotypic information.^2^ AMCs are in a unique position to collect biospecimens as patients share diverse specimen types and related phenotypic information as part of their clinical care. In fact, hospitals are being “inundated with requests” from industry across the country.^3^

Industry is also a key component of financially supporting AMC biobanks.^4^ In recent years, types of data remuneration arrangements have expanded to licensing agreements for industry-derived machine-learning models, discounted clinical data analyses, electronic medical record “awards” and invoice credits, or academic advantages such as access to data necessary for high-impact research.^5^ For example, in *Dinerstein v Google*,^6^ employees from Google and UChicago published the AI model, trained on UChicago patients, together.^7^

But as Schwartz and Hartsock explore, many patients when asked are uncomfortable with specimen commercialization.^8^ Looking at health information, patients also report they are particularly uncomfortable with data sharing for profit and are concerned that profit-driven users might burden or exploit patients.^9^

So, how can we respect patient autonomy interests while maintaining the public benefit of enabling industry/AMC research collaboration? As asked by the authors: do AMCs owe their patients something more than required by law? And if so — what is it?

## Solution 1: Ask Patients for their Permission

As highlighted by Schwartz and Hartsock, the first potential solution to the above problem is for AMCs to ask patients for their permission to commercialize their biospecimens. This, theoretically, would ensure that all commercialized biospecimens came from fully consenting individuals, ensuring respect for their autonomy. But there are two problems with this solution.

First, *it limits the impact of research with biospecimens*. As argued by Neil Richards and Woodrow Hartzog, “consent does not scale.”^10^ To request and store (and potentially withdraw) individual consent for each specimen contribution is prohibitively expensive and unfeasible for the number of specimens necessary for research.^11^ It can also limit the demographic variation of the specimen contributor cohort. Differences in consent rate can be associated with self-identified race, ethnicity, and socioeconomic status.^12^ A lack of demographic variation can be important for genetic research, insofar as self-identified race and ethnicity is associated with genetic ancestry, and is sometimes used as a proxy-indicator for other social determinants of health (although this use is being critically reevaluated).^13^

Second, as authors and others have demonstrated elsewhere, *asking patients for their informed consent to commercialization does not generally increase their knowledge or understanding*.^14^ While these two problems appear to be in conflict (how can asking for informed consent for commercialization impact consent but not knowledge?), the conflict is likely due to study design. Having a fulsome conversation with a researcher about specimen commercialization increases knowledge and decreases hypothetical consent, but just being given a consent to sign which includes a commercialization disclosure — as a patient would during normal clinical care — does not increase knowledge or impact consent.

But what are the actual implications of such transparency? If patients realize that their specimens were commercialized, will the fact that it was disclosed previously stabilize trust? Will it negatively impact patient clinical behavior? Will it further marginalize or limit the options of historically excluded patient populations? These are critical questions that need to be answered as we move forward.

## Solution 2: Maintain the Status Quo

The simpler solution is the preferred one for most AMCs in the country: adhere to the law and move on. This position is not without ethical merit, the law was developed with extensive and thoughtful debate. The federal government spent six years publicly discussing the ethical issues at stake in requiring consent for all biospecimen research. While they acknowledged requiring consent for de-identified biospecimen research appeared “consistent with the majority of the public’s wishes,” they ultimately argued that such a requirement would “allow autonomy to trump beneficence and justice” as, among other things, “it would result in fewer specimens collected from fewer sources, with adverse implications for rare diseases and for justice…”^15^ Faden et al. also classically argued in 2013 that patients can even be seen as having a moral *obligation* to participate in low-risk data research to improve health care as part of a learning health system from which they too benefit.^16^

Critically, however, both the federal government and Faden et al. were talking about research generally — not the commercialization of biospecimens specifically. And patients are more concerned about notice regarding commercial versus academic research. For example, in a recent survey, we found that the majority of patients (n=2054) reported that it was “very true” they were interested in notification regarding commercial use of their biospecimens (both identified and deidentified) and were *more likely* to want notice regarding commercial use versus university use.^17^

## What’s Behind Door #3?

I agree that patient autonomy interests in *consent* to low-risk biospecimen research are generally outweighed by the public interest in supporting clinical research. But, given that 1) patients are even more likely to want notice regarding commercial versus university use of biospecimens and 2) not all commercial biospecimen research actually contributes to the public good, I think it is hard for AMCs to justify a lack of *notice*. Schwartz and Hartsock also point out the critical function of patient trust,^18^ which can be decreased when patients are surprised about biospecimen commercialization.

But does disclosure add ethical value if patients are not going to understand (or even read) what is written on the form? Are AMCs being more respectful of patients if they give them notice, even without an opportunity to opt out? I agree with authors that the answer is yes.^19^ At Michigan Medicine we are currently refining a model for how to do so.^20^ The majority of patients want notice, and disclosure without an opt-out is unlikely to impact the number of research specimens. That is an obvious compromise to make between individual autonomy and public good.

But what are the actual implications of such transparency? If patients realize that their specimens were commercialized, will the fact that it was disclosed previously stabilize trust? Will it negatively impact patient clinical behavior? Will it further marginalize or limit the options of historically excluded patient populations? These are critical questions that need to be answered as we move forward.
